# Infant and Young Child Feeding Practices among Adolescent Mothers and Associated Factors in India

**DOI:** 10.3390/nu13072376

**Published:** 2021-07-12

**Authors:** Mansi Vijaybhai Dhami, Felix Akpojene Ogbo, Thierno M. O. Diallo, Bolajoko O. Olusanya, Piwuna Christopher Goson, Kingsley Emwinyore Agho, on behalf of the Global Maternal and Child Health Research Collaboration (GloMACH)

**Affiliations:** 1 Translational Health Research Institute (THRI), School of Medicine, Campbelltown Campus, Western Sydney University, Penrith, NSW 2571, Australia; f.ogbo@westernsydney.edu.au (F.A.O.); k.agho@westernsydney.edu.au (K.E.A.); 2 Barmera Medical Clinic (Lake Bonney Private Medical Clinic), Barmera, SA 5345, Australia; 3 School of Social Sciences, Western Sydney University, Penrith, NSW 2571, Australia; t.diallo@westernsydney.edu.au; 4 Statistiques & M. N., Sherbrooke, QC J1K 2Z4, Canada; 5 Centre for Healthy Start Initiative, 286A Corporation Drive, Dolphin Estate, Ikoyi, Lagos 101233, Nigeria; bolajoko.olusanya@uclmail.net; 6 Department of Psychiatry, College of Health Sciences, University of Jos, Jos 930003, Nigeria; piwunag@unijos.edu.ng; 7 African Vision Research Institute (AVRI), University of KwaZulu-Natal, Durban 4041, South Africa; 8 School of Health Sciences, Campbelltown Campus, Western Sydney University, Penrith, NSW 2571, Australia

**Keywords:** infant and young child feeding, breastfeeding, complementary feeding, adolescent mothers, India

## Abstract

Adequate infant and young child feeding (IYCF) improve child survival and growth. Globally, about 18 million babies are born to mothers aged 18 years or less and have a higher likelihood of adverse birth outcomes in India due to insufficient knowledge of child growth. This paper examined factors associated with IYCF practices among adolescent Indian mothers. This cross-sectional study extracted data on 5148 children aged 0–23 months from the 2015–2016 India National Family Health Survey. Survey logistic regression was used to assess factors associated with IYCF among adolescent mothers. Prevalence of exclusive breastfeeding, early initiation of breastfeeding, timely introduction of complementary feeding, minimum dietary diversity, minimum meal frequency, and minimum acceptable diet rates were: 58.7%, 43.8%, 43.3%, 16.6%, 27.4% and 6.8%, respectively. Maternal education, mode of delivery, frequency of antenatal care (ANC) clinic visits, geographical region, child’s age, and household wealth were the main factors associated with breastfeeding practices while maternal education, maternal marital status, child’s age, frequency of ANC clinic visits, geographical region, and household wealth were factors associated with complementary feeding practices. IYCF practices among adolescent mothers are suboptimal except for breastfeeding. Health and nutritional support interventions should address the factors for these indicators among adolescent mothers in India.

## 1. Introduction

Globally, appropriate infant and young child feeding (IYCF), comprising breastfeeding (BF) and complementary feeding (CF) practices, play an essential role in child growth, development, and survival [1]. In 2015, approximately 6 million deaths under five years old were reported, and more than 67% of these deaths were attributed to inappropriate child feeding practices [2,3]. Inappropriate feeding practices are behaviors that are inconsistent with recommended IYCF. The key recommended IYCF behaviors include the initiation of breastfeeding for newborns within the first hour of birth, exclusive breastfeeding for the first 6 months of life and continued breastfeeding for two years and beyond with nutritionally appropriate and safe complementary foods introduced during the sixth month [2,4].

Several factors are known to be associated with breastfeeding practices; these include economic status [5], maternal education [6], employment status [7], residence type [8], mode and place of birth delivery [9], infant feeding counseling, and sex and age of the child [8]. Despite that these factors affect women of all reproductive ages, it is evident that adolescent mothers are more physiologically and socioeconomically disadvantaged, which may result in a higher prevalence of suboptimal breastfeeding practices and worse health outcomes among their children [10]. Adolescent and young mothers are less likely to initiate breastfeeding [10], more likely to prematurely discontinue exclusive breastfeeding [11], and breastfeed for a shorter overall duration [12] compared with their older counterparts. Furthermore, the health outcomes of the children of these adolescent mothers are comparably worse than those born to older mothers [13]. These issues explain why adolescents have become an important population group in global efforts to achieve equitable health through the Leave No One Behind initiative to achieve the Sustainable Development Goals (SDGs), numbers 2 and 3, by 2030 [14].

There are several issues that highlight the significance of exploring and understanding breastfeeding practices among adolescent mothers. First, although adolescent fertility rates have declined worldwide, they remain high in many low- and middle-income countries. In 2016, there were more than 11 million live births among adolescent mothers aged between 15 and 19 years [15], and a considerable number of these infants did not receive optimal breastfeeding. Second, because breastmilk can mitigate or offset some of the social and economic disadvantages faced by adolescent mothers and their infants, research and interventions geared toward this population’s specific needs and concerns are critically required. This may be because adolescent mothers have unique challenges and vulnerabilities that make them substantially different from older mothers, which result in specific concerns about breastfeeding practices [16].

Despite that appropriate CF among children aged 6–23 months brings several health benefits [17], the inappropriate introduction of CF practices may increase the likelihood of malnutrition among children under five years [18]. Levels of CF may be affected by several individual-, household- and community-level factors [19]. Undernourished children have a higher likelihood of developing severe health hazards that can impede the body’s metabolism and retard utilization of immunity due to deficiencies in immune competence [20].

A systematic review conducted for India in 2017 indicated that CF behaviors were largely influenced by limited knowledge of appropriate CF practices and low parental education [20], among others. There is limited knowledge of appropriate IYCF practices among adolescent mothers [21]. Consequently, and as there has not been any previous research on IYCF practices and adolescent mothers in India, this current study sought to examine factors associated with IYCF practices among adolescent Indian mothers using the 2008 recommended IYCF indicators [22]. The study can be of immense benefit to regional development assistance partner establishments to allocate resources, as well as evaluate nutrition-sensitive interventions and programs in order to reduce child deaths triggered by inadequate IYCF practices among adolescent mothers.

## 2. Methods

### 2.1. Data Sources and Study Design

This study utilized data extracted from the 2015–2016 India National Family Health Survey (NFHS-4); also referred to as the 2015–16 India Demographic and Health Survey (DHS), conducted by the International Institute for Population Sciences, Mumbai, India. Details of the methodology and sampling procedure of the survey can be found elsewhere [23]. Sociodemographic, household characteristics, and data on infant and young child feeding practices were collected from a sample of respondents (adolescent mothers aged between 15 and 19 years). A multistage cluster sampling design was used for the survey (which adopted a standardized questionnaire), from NFHS-4. The study was limited to children who were alive, of singleton births, last-born, aged 0–23 months and lived with the respondent. The survey yielded a weighted total of 5148 children with an average response rate of 94%.

### 2.2. Outcome and Confounding Factors

The study outcomes were the 2008 IYCF (BF and CF) indicators prescribed by the World Health Organization (WHO) [24]. The study was based on the recall of mothers regarding the food they fed their child within the 24 h preceding the survey. In this study, we considered four key BF indicators because exclusive breastfeeding and early initiation of breastfeeding are protective factors for child mortality and morbidity, while predominant breastfeeding and bottle feeding increases the risk of diarrhea and respiratory illness [25]. 

The selected BF indicators for our study are defined below:

- Exclusive breastfeeding under 6 months: the proportion of infants 0–5 months of age who received breast milk during the previous day;
- Predominant breastfeeding under 6 months: the proportion of infants 0–5 months of age who received breast milk as the predominant source of nourishment during the previous day;
- Early initiation of breastfeeding: the proportion of children born in the last 24 months who started breastfeeding within one hour of birth;
- Bottle feeding: the proportion of Children 0–23 months of age who were fed with a bottle during the previous day;

The CF indicators considered in the study are defined below:

- Introduction to solid, semisolid or soft foods: the proportion of infants 6–8 months of age who received solid, semisolid or soft foods during the previous day;
- Minimum dietary diversity: the proportion of Children 6–23 months of age who received foods from ≥4 food groups during the previous day. The seven food groups considered were (1) grains, roots, and tubers; (2) legumes and nuts; (3) dairy products (milk, yogurt, and cheese); (4) flesh foods (meat, fish, and poultry); (5) eggs; (6) vitamin A-rich fruits and vegetables; and (7) other fruits and vegetables;
- Minimum meal frequency: the proportion of breastfed children 6–23 months of age who received solid, semisolid, or soft foods the minimum number of times or more during the previous day, and the proportion of non-breastfed children 6–23 months of age who received solid, semisolid, or soft foods or milk feeds the minimum number of times or more during the previous day;
- Minimum acceptable diet: the proportion of breastfed children 6–23 months of age who had at least the minimum dietary diversity and the minimum meal frequency during the previous day and the proportion of non-breastfed children 6–23 months of age who received at least two milk feedings and had at least the minimum dietary diversity, not including milk feeds and the minimum meal frequency during the previous day.

The independent variables were composed of socio-demographic and economic characteristics of children and their parents. The choice to use these variables was informed by previous literature [26,27,28,29] and their availability in the India 2015–2016 NFHS dataset. They were classified into three levels: individual-, household- and community-level factors. Individual-level factors consisted of characteristics of the mother (religion, age, work status, education, literacy, body mass index (BMI), age, marital status, place and mode of delivery, delivery assistance, number of antenatal visits, postnatal checks, access to the media, and power over earning and decision making), the father (occupation) and the child (sex, age, birth weight, birth order, birth interval, illness, and perceived size at birth). The wealth index, number of living children, quality of the source of drinking water, and quality of the type of toilet facility constituted the household-level factors, while the community-level factors were composed of the type of residence, geographic region, and type of caste or tribe. In the NFHS, the principal components analysis [30] was used to construct the household wealth index. The latter was calculated as a score of ownership of household assets, such as transportation device, ownership of durable goods, and household facilities. Furthermore, the index was divided into three categories, namely, poor, middle, and rich (detailed information on the definition and categorization of potential confounding variables used in the study is provided in Supplementary Table S1).

### 2.3. Statistical Analysis

The strategy for the analyses in this study was in line with that of previously published research [31,32,33]. Preliminary analyses involved the assessment of frequencies and cross-tabulations to estimate the prevalence of all the IYCF indicators used in the study. An estimation of the prevalence and corresponding confidence intervals of IYCF indicators then followed. IYCF indicators used in the study were categorized as binary (yes as ‘1′ and no as ‘0′) and we then conducted univariable and multivariable survey logistic regression analyses to examine the association between the study variables (individual-, household- and community-level factors) adjusted for clustering and sampling weights. 

Survey logistic regressions that adjusted for cluster and survey weights were used to identify unadjusted odds ratios of all the study outcomes. A three-staged modelling technique was adopted for the survey multivariable analyses in which level factors were entered progressively into the model to assess associations with the study outcomes. In the first stage, individual-level factors were entered into the baseline multivariable model to examine their association with the study outcomes. Thereafter, a manually executed elimination method was used to determine factors associated with IYCF at a 0.05 significance level (Model 1). In the second stage, household-level factors were added to Model 1, and those factors with *p*-values < 0.05 were retained (Model 2) after a manually executed elimination method was conducted. In the third stage, community-level factors were added to Model 2. As before, those factors with *p*-values < 0.05 were retained (Model 3). Only those factors significantly associated with IYCF at a 5% significance level in Model 3 were reported in the study.

In the final model, we tested and reported any co-linearity. We then calculated the odds ratios with 95% confidence intervals derived from the adjusted logistic regression models, which were used to determine the level of association of the factors of possible confounding variables, and all analyses were conducted using Stata version 14.0 (Stata Corp, College Station, TX, USA).

## 3. Results 

### 3.1. Characteristics of the Study Population

Table 1 demonstrates the individual-, household-, and community-level characteristics of the study population included in this analysis. Nearly 8.5% of adolescent mothers were employed in the past 12 months prior to the survey and more than two-thirds (69.6%) had secondary education or higher. Approximately more than half of adolescent mothers (57.1%) were from poor households, and the majority (80.7%) were from rural areas. Of the respondents, 71% never read newspapers, 88.6% never listened to the radio, and 28.7% never watched television. Low body mass index (BMI) (<18.5 kg/m^2^) was observed in 27.7% of adolescent mothers; 16.6% of mothers did not attend an antenatal clinic (ANC) during pregnancy, and 72.3% of them had health professionals available at delivery. 

### 3.2. Breastfeeding and Infant Feeding Indicators

As shown in Table 2, of the total 5148 children aged 0–23 months, early initiation of breastfeeding was observed in only 43.8%, and the exclusive breastfeeding rate in children aged less than 6 months was 62% and ever breastfed rates was 61.9%. The breastfeeding continuation in the first year was 88.2% and in the second year was 71.3%. Bottle feeding and predominant breastfeeding rates were 16.6% and 17.8%, respectively. Early initiation of complementary feeding was 43.3%, whereas minimum dietary diversity, minimum meal frequency, and minimum acceptable diet rates were 16.6%, 27.4%, and 6.8%, respectively.

**Figure 1:**
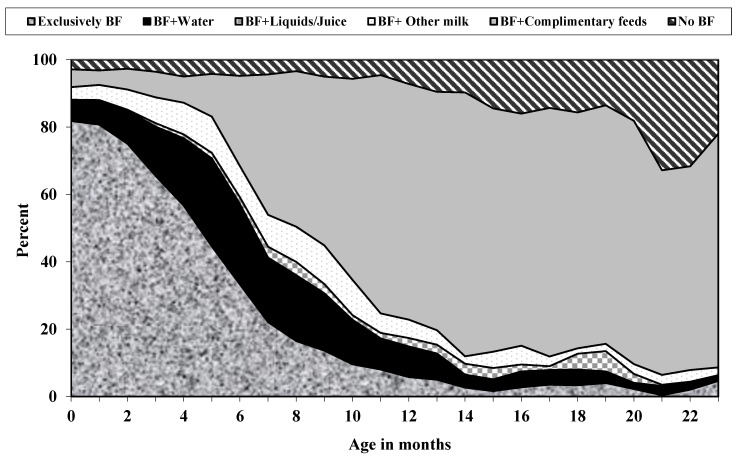
Distribution of children by breastfeeding (BF) status, according to age among adolescent mothers in India, 2015–2016.

According to age, the proportion of children born to adolescent mothers in India by breastfeeding status in 2015–2016 is presented in Figure 1. Exclusive breastfeeding at birth was near 82%, which declined to 61% by the third month. At birth, the proportion of infants breastfed, in addition to water, was 6.3%, which increased to about 11.5% at 2 months. Similarly, infants breastfed, in addition to other milk, was 3.4% at birth, which increased to 5.2% by 2 months of age. The median duration of exclusive breastfeeding and any breastfeeding were 4.6 and 23.0 months, respectively.

### 3.3. Factors Associated with Breastfeeding Indicators

Table 3 and Table 4 reported factors associated with key breastfeeding indicators. The factors associated with early initiation of breastfeeding and exclusive breastfeeding as shown in Table 5, and factors associated with bottle feeding and predominant breastfeeding among adolescent mothers are reported in Table 4.

### 3.4. Early Initiation of Breastfeeding

Adolescent mothers who had vaginal births (adjusted odds ratio (OR): 1.36; 95% confidence interval (CI): 1.06, 1.73) and those who resided in the northeast, south, east, and west regions were more likely to introduce breastfeeding within 1 h of life compared with the north region (Table 3). The odds of not engaging in early initiation of breastfeeding were lower in adolescent mothers who did not attend ANC (OR: 0.58; 95% CI: (0.41, 0.83)). Infants with diarrhea and fever two weeks prior to the survey were more likely to have a delayed introduction to breastfeeding within 1 h of life.

### 3.5. Exclusive Breastfeeding

Mothers who belonged to scheduled tribes were significantly associated with increased odds of exclusive breastfeeding among adolescent mothers (OR: 2.00; 95% CI: 1.27, 3.16). Factors significantly associated with decreased odds of exclusive breastfeeding among adolescent mothers were: primary education (OR: 0.55; 95% CI: 0.34, 0.88), 2nd–4th born infant (OR: 0.46; 95% CI: 0.31, 0.66), central region (OR: 0.63; 95% CI: 0.42, 0.95) and diarrhea in the two weeks preceding the survey (OR: 0.62; 95% CI: 0.40, 0.95).

### 3.6. Bottle-Feeding

Factors significantly associated with increased odds of bottle feeding among adolescent mothers were: 12–17 month age group (OR: 2.07; 95% CI: 1.52, 2.80), caesarean birth (OR: 2.45; 95% CI: 1.58, 3.81), listening to radio (OR: 1.51; 95% CI: 1.07, 2.11), rich household (OR: 1.61; 95% CI: 1.20, 2.16) fever two weeks preceding the survey (OR: 1.54; 95% CI: 1.17, 2.03) (Table 4). Belonging to a scheduled tribe was significantly associated with decreased odds of bottle feeding (OR: 0.61; 95% CI: 0.40, 0.92).

### 3.7. Predominant Breastfeeding

Adolescent mothers who completed primary education and infants delivered by a friend, relative, or others were more likely to predominantly breastfeed their infants. Predominant breastfeeding increased as child age increased (OR: 1.51; 95% CI: 1.35, 1.69). Adolescent mothers who lived in the northeast were less likely to predominantly breastfeed their infants than those who lived in the north region.

### 3.8. Factors Associated with Complementary Feeding Indicators

The factors associated with introduction to solid, semisolid, or soft foods and minimum dietary diversity are presented in Table 5, while Table 6 presents factors associated with minimum meal frequency and minimum acceptable diet among adolescent mothers.

### 3.9. Introduction to Solid, Semisolid, or Soft Foods 

Middle wealth index households (OR: 2.57; 95% CI: 1.50, 4.40) and living in the central, south, west and northeast regions were significantly associated with increased odds of introduction to solid, semisolid, or soft foods. Decreased odds of introduction to solid, semisolid, or soft foods were significantly associated with having attended between one and three ANC clinics (OR: 1.54; 95% CI: 1.17, 2.03).

### 3.10. Minimum Dietary Diversity

Factors significantly associated with adequate minimum dietary diversity among adolescent mothers were: secondary education and higher (OR: 1.67; 95% CI: 1.10, 2.52), 18–23 and 12–17 months age group, living in the south, east, and northeast regions, and fever in the two weeks preceding the survey (OR: 1.65; 95% CI: 1.09, 2.48). Factors significantly associated with decreased odds of meeting minimum dietary diversity were: formerly married (divorced, separated, or widowed) (OR: 0.19; 95% CI: 0.06, 0.60), attended no ANC clinics (OR: 0.47; 95% CI: 0.26, 0.84), and other backward caste (OR: 0.64; 95% CI: 0.42, 0.98).

Minimum meal frequency factors significantly associated with increased minimum meal frequency among adolescent mothers were: mothers aged 18 and 19 years, primary education, secondary or more education, and living in the central and east regions. Other backward caste was significantly associated with decreased odds of minimum meal frequency (OR: 0.73; 95% CI: 0.54, 0.98).

### 3.11. Minimum Acceptable Diet

Factors significantly associated with increased odds of meeting minimum acceptable diet among adolescent mothers were: secondary education or higher (OR: 1.94; 95% CI: 1.06, 3.53) and living in the south, east, and northeast regions. Formerly married (separated, divorced, or widowed) (OR: 0.12; 95% CI: 0.03, 0.44) and other backward caste factors were significantly associated with decreased odds of meeting a minimum acceptable diet.

## 4. Discussion

The current study revealed that IYCF (BF and CF) practices, except for ever breastfed among adolescent mothers, were suboptimal and below the target of 90% coverage. The study population’s characteristics that were significantly associated with IYCF indicators included maternal level of education, maternal marital status, child’s age, number of ANC clinic visits, geographical region, caste or tribe, and child illness.

Our analysis revealed that early initiation of breastfeeding rates among adolescent mothers were 43.8%, which are slightly higher than that of Bangladesh (42.2%), regarding the same survey [34]. The suboptimal early initiation of breastfeeding among adolescent mothers needs improvement to enhance the infant’s survival and welfare. Our analysis also showed that the rate for introduction to solid, semisolid, or soft foods among adolescent mothers was 43.3%. Around the same survey period, the corresponding rate for Bangladesh was 64.9% [34], indicating that India reported a much poorer timely introduction of CF than Bangladesh. This suggests that adequate CF practices remain a challenge in Indian adolescent mothers. Therefore, studies to understand factors affecting CF practices among Indian adolescent mothers are prudent, and this study contributes to that knowledge.

### 4.1. Breastfeeding Indicators

We found in this study that giving birth by caesarean section was associated with decreased odds of early initiation of breastfeeding among adolescent mothers. This finding is consistent with a past study conducted in Nigeria that observed that infants born by caesarean section had 87% lower odds of early initiation of breastfeeding compared with those born vaginally [35]. This finding can be explained by the fact that caesarean section birth lowers the odds of early initiation of breastfeeding as a result of factors that include prolonged separation of the mother and her baby, surgical issues such as the effect of anesthesia, and the stress or fatigue due to complicated labor or other birth complications [21].

This study found that adolescent mothers who did not attend ANC clinics were significantly more likely to delay breastfeeding initiation. This finding is corroborated by previous other studies from Nigeria [34], Bangladesh [36], and India [37]. However, previous studies have revealed a negative association with the number of ANC clinic visits and early initiation of breastfeeding [21], and despite the counterintuitive nature of this finding, it is believed that frequent ANC clinic visits may be a sign of increased care contact as a result of potentially complicated pregnancies in which there is the likelihood of women experiencing ANC and intrapartum challenges that require medical intervention [35].

Our study found that children who had diarrhea were significantly more likely to experience delayed initiation of breastfeeding compared with their counterparts who did not suffer from it. A study conducted from India [25] corroborates this finding by showing that early initiation of breastfeeding was a protective factor for child diarrhea in this country. The finding has significant policy implications for current and future efforts to reduce the burden of diarrheal disease in Indian children. Consequently, it is suggested that current breastfeeding programs in India, like the Mother’s Absolute Affection program [38], which aims to create support, and generate and provide an enabling environment for mothers, family members, and community, should also consider providing information to mothers and their families about the additional benefits of optimal breastfeeding, including prevention of diarrheal disease in children.

Our study revealed that children of adolescent mothers who had fever during the fortnight preceding the survey were significantly more likely to delay initiation of breastfeeding compared to those who did not have the disease, which is consistent with the results of a past study from Bangladesh and highlights the importance of encouraging women and caregivers to facilitate early initiation of breastfeeding in order to reduce early newborn danger signs and severe illnesses, especially among high-risk newborns.

Our study found that predominant breastfeeding increased as infant age increased and decreased among adolescent mothers who reside in the northeast region. These findings were consistent with a cross-sectional study conducted in Bangladesh, which revealed that the odds of predominant breastfeeding were higher among infants under 6 months of age and lower among adolescent mothers residing in all regions except the Barisal region [34]. The odds of predominant breastfeeding were higher among Adolescent mothers with completed primary education and those delivered by a friend, relative, or others. These findings are supported by a cross-sectional study conducted in Kenya, which revealed that over 52% of adolescent mothers were delivered by unskilled birth attendance and those who completed primary education were more likely to be delivered by unskilled birth attendance [39]. Past studies also indicated the effect of traditional beliefs, in which mothers-in-law and elders believe that giving water to infants during exclusive breastfeeding practices helps with cleaning the infant’s intestines [8,40].

In this study, adolescent mothers who were educated up to primary school were significantly less likely to practice exclusive breastfeeding versus their counterparts with no schooling. This finding is contrary to findings of several other studies that show that low levels of maternal education are associated with a lower prevalence of exclusive breastfeeding in the first six months of life [5]. Findings from these past studies may be explained by mothers with a higher education level have more exposure to various sources of information and better knowledge about appropriate infant and young child feeding compared to uneducated mothers. Hence, mothers with a lower level of education should be given extra support to maintain exclusive breastfeeding until 6 months.

We found that 2nd–4th born children of adolescent mothers were significantly less likely to have exclusively breastfed compared with 1st born children, indicating that younger children were more likely to be exclusively breastfed compared with older ones. This finding is corroborated by past studies [34], which observed that exclusive breastfeeding was more likely in younger children than in older ones and may be attributed to the fact that the child’s age increases as exclusive breastfeeding decreases.

Children of adolescent mothers who suffered from diarrhea were significantly less likely to have been exclusively breastfed compared with their counterparts who did not suffer from the disease. This negative association between diarrhea and Exclusive breastfeeding is consistent with a past study [41]. Similar findings were made in previous studies [42]. Children who did not receive exclusive breastfeeding are likely to be fed other complementary foods, which are primary sources of gastrointestinal pathogens [43] and which may be the most probable explanation for this observed association.

Children of adolescent mothers 12–17 months of age were found to be significantly positively associated with the bottle feeding rate compared with their younger counterparts in the 0–5 month age bracket, indicating that older children were more likely to be bottle-fed versus younger children. This finding is consistent with that of a previous study from Indonesia [44], where older children were associated with bottle feeding relative to their younger counterparts. This may be explained by the fact that as a child’s age increases, they are likely to have feeding alternatives, which can increase the rate of feeding bottle usage. The use of bottle feeding as children grow older may be explained by the use of the bottle with an intake of water, tea and processed milk, which are commonly given as the age of the child increases [44].

Our study observed that compared with children of adolescent mothers born through non-caesarean section, those born through caesarean section were significantly more likely to be bottle fed. This is in accordance with a past study from Indonesia [44], which observed that mothers who had caesarean births were more likely to bottle feed than to breastfeed, compared with mothers who had vaginal deliveries. Our finding also aligns with another past study that recorded 15% of mothers with health-facility caesarean births used bottle feeding [45]. A previous study indicated that women who delivered through caesarean section assumed that they did not have sufficient breast milk, which prompted them to resort to bottle feeding [42].

The odds of offering bottle feeding to adolescent mothers were significantly higher among adolescent mothers from rich households than those from poor households. This result implies that infants born to adolescent mothers from poor households were less likely to be bottle fed. This finding conforms with that from a past cross-sectional study conducted in Indonesia that observed that rich households reported an increased likelihood of bottle feeding [46]. The significantly lower likelihood of bottle feeding among adolescent mothers from poor households reported in our study may be due to the inability to finance or have access to infant formula or expensive complementary substitutes to breast milk, which may also cause poor adolescent mothers to depend fully on breastfeeding [44]. Other previous studies observed that the association between wealth and bottle feeding is possibly related to a rich household’s ability to access breastfeeding alternatives, such as the use of nipple or bottle feeding [45]. Family income has also been attributed to the positive contribution toward the practice of bottle feeding among adolescent mothers [47]. One strategy in addressing this issue is to establish educational campaigns at all levels of society irrespective of economic status.

The likelihood of practicing predominant breastfeeding was significantly higher among adolescent mothers from the northeast region versus their counterparts from the north region. The finding of regional differences in practicing predominant breastfeeding by adolescent mothers is consistent with past studies [34]. Additionally, a past study from Bangladesh [34] revealed that mothers living in Chittagong, Dhaka, and Rajshahi reported a lower prevalence of predominant breastfeeding rates than those in Sylhet.

### 4.2. Complementary Feeding Indicators

Adolescent mothers who had a minimum of 1–3 ANC clinic visits were found to be significantly less likely to meet the requirement for introduction to solid, semisolid, or soft foods compared with their counterparts who had a minimum of eight ANC clinic visits, indicating that increased odds of practicing introduction to solid, semisolid, or soft foods was associated with higher ANC clinic visits. This is consistent with the results for Sierra Leone and Liberia in a past study, which examined suboptimal CF practices in four Anglophone West African countries [48] and also in past studies from India [49] and Bangladesh [50]. This finding may be attributed to the cost associated with attending ANC clinics and a lack of education and awareness of the importance of ANC among mothers [51,52,53]. Thus, effective nutrition education and counselling, which are normally provided at ANC clinics, are necessary for many communities in India. It is suggested that such counseling should focus on vulnerable regions and populations so as to maximize the impacts of nutritional interventions such as improving infant feeding knowledge and practices of mothers [54].

In this study, we found that the odds of meeting the requirement for introduction to solid, semisolid, or soft foods were significantly higher among adolescent mothers from medium wealth households than those from poor households, indicating that the requirement for introduction to solid, semisolid, or soft foods is a function of affluence. This is in accordance with a past study from Bangladesh [55] that revealed that children from socioeconomically middleclass households were less likely to receive adequate CF as compared with rich children. The results suggest that richer households can afford the cost of complementary foods for their children. Despite the numerous policies and strategies that have been issued in India to improve IYCF, there still exists challenges such as insufficient resources and lack of coordination among stakeholders, which impede their implementation and enforcement. Consequently, the Indian government and other stakeholders should consider strengthening existing strategies; for example, eradicating poverty through marginalized and vulnerable group development, empowering mothers to practice decision making autonomy, and minimizing rural-urban differentials by planning and providing modern facilities to improve of the situation of CF in India.

We found that adolescent mothers living in the central region were significantly more likely to practice introduction to solid, semisolid, or soft foods versus their counterparts from the north region. These findings are consistent with a past study from Malawi [56], in which a multivariate analysis revealed that mothers who resided in the country’s central region had increased odds of introduction to solid, semisolid, or soft foods compared with those from their northern region. The findings suggest that adolescent mothers living in particular regions may have higher socioeconomic status, which is a determinant of introduction to solid, semisolid, or soft foods.

Our study revealed that maternal education was positively associated with adequate complementary feeding practices. Adolescent mothers who attained secondary education or higher were significantly more likely to meet the requirements for minimum dietary diversity than their counterparts with no schooling. Adolescent mothers who had primary education were significantly more likely to provide minimum meal frequency than those without schooling, consistent with a past study from southern Ethiopia [57], which found illiterate mothers were less likely to feed their children to meet the requirement for minimum meal frequency versus their literate counterparts. Furthermore, adolescent mothers with a secondary education or higher had higher odds of meeting the minimum acceptable diet requirement than those who had no schooling. These findings are consistent with findings from other past studies. For instance, a study from Malawi [56] observed that children whose mothers with primary education or secondary and post-secondary education were more likely to achieve minimum dietary diversity. The findings may be due to educated women being more likely to have access to quality health services and messages, which they may more easily comprehend and apply [58].

Adolescent mothers who were not married were significantly less likely to meet the requirement for minimum dietary diversity, and significantly more likely to meet the minimum acceptable diet requirement versus their married counterparts. The association of unmarried mothers with minimum dietary diversity is consistent with a previous study from Ethiopia [59]. This finding is plausible, as married mothers living with their husbands have been more knowledgeable in dietary diversity for children [59] while husbands contribute money to procure different foods. Adolescent mothers should be encouraged to stay in relationships to receive the necessary support from their husbands. Widowed mothers should be encouraged to remarry.

In our study, it was found that the likelihood of meeting the requirement for minimum dietary diversity was significantly higher among children who belonged to the 12–17 months age bracket, compared with those belonging to the 0–5 months age bracket, indicating that increased age was positively associated with minimum dietary diversity. This finding is similar to that of a past study from Indonesia [60], in which children aged 12–17 months were more likely to receive various foods with each meal than younger children. This finding should motivate health workers in India to promote the benefits of various foods, targeting mothers with younger children.

Adolescent mothers who did not attend any ANC clinics were significantly less likely to meet the requirement for minimum dietary diversity, consistent with a study from Ethiopia [61]. Mothers who have a high frequency of ANC clinic visits may be more informed, have greater access to services, and may be from a wealthy family, and thus are more likely to afford and provide a variety of foods to their children more frequently.

An important strength of our study was that it utilized data from the 2015–2016 India NFHS, which is nationally representative and population based, and therefore results from it can be generalized for the Indian population. The study, however, had several limitations. First, minimum dietary diversity was defined as the proportion of children aged 6–23 months who consumed ≥4 of 7 food groups (minimum dietary diversity—7) in the previous 24 h instead of ≥5 of 8 food groups (minimum dietary diversity—8) in the previous 24 h as reported [62], but a current study that examines minimum dietary diversity in eastern and southern African regions indicated that confidence intervals for prevalence estimates regarding minimum dietary diversity—7 and minimum dietary diversity—8 overlapped in most eastern and southern African countries [63]. Second, this study did not consider the new IYCF indicators such as mixed milk feeding, sweet beverage consumption, unhealthy food consumption, and zero vegetable or fruit consumption, which was reported in the 2020 IYCF indicators [62], Third, several discussed factors associated with IYCF practices may differ for adolescent and adult mothers. Last, due to the cross-sectional nature of the survey design, we could not establish any cause and effect relationships. 

## 5. Conclusions

Our study has shown key issues that need to be addressed regarding adolescent Indian mothers and IYCF practices. Maternal age in relation to breastfeeding issues has often been explored as a confounding factor in previous studies yet rarely as the primary research interest. This study is therefore exceptional, as it focused on adolescent mothers as the main research interest. 

Our findings indicate the need to set a future research agenda around developing comprehensive individual-, household-, and community-based interventions focused on promoting protective breastfeeding and complementary feeding practices. At the household level, educational support intervention is needed and should focus on providing favorable conditions for adolescent mothers to re-enter school after giving birth because, in many lower and middle income countries (LMIC), including India, adolescent pregnancy is considered the woman’s fault. Community-based IYCF intervention programs are needed to improve the health and nutrition of adolescent mothers and their babies by discouraging inadequate IYCF practices in India, including addressing maternal healthcare services in disadvantaged regions.

## Figures and Tables

**Table 1:** Characteristics of the study population, India (*n* = 5148).

Characteristic	*n*	%
***Individual-level factors***		
**Mother’s religion (*n* = 5147)**		
Hindu	3997	77.7
Muslim	913	17.7
Christian and others	237	4.6
**Mother’s working status**		
Nonworking	5071	98.5
Working (past 12 months)	76	1.5
**Mother’s education**		
No education	936	18.2
Primary	631	12.3
Secondary and above	3581	69.6
**Father’s occupation**		
Nonagricultural	527	10.2
Agricultural	311	6.0
Not working	4310	83.7
**Total Maternal BMI (Kg/m^2^_,_*n* = 5083)**		
<18.5	1408	27.7
18.5–24.9	3382	66.5
25+	293	5.8
**Mother’s age (years)**		
15–17	668	13.0
18	1650	32.1
19	2830	55.0
**Marital status (*n* = 5135)**		
Currently married	5091	99.1
Formerly married ^#^	44	0.9
**Combined Mode and Place of delivery**		
Non-caesarean and home	759	14.8
Non-caesarean and health facility	3517	68.3
Caesarean and health facility	871	16.9
**Type of delivery assistance**		
Health professional	3704	72.4
Traditional birth attendant	450	8.8
Others ^&^	966	18.9
**Number of antenatal clinic visits (*n* = 4885)**		
8+	761	15.6
4–7	1806	37.0
1–3	1532	31.4
None	786	16.1
**Timely postnatal check**		
0–2 days	122	2.4
After 2 days	239	4.7
No postnatal check	4787	93.0
**Mothers reading newspapers**		
Not at all	3659	71.1
Yes/some ^	1489	28.9
**Mothers listening to radio**		
Not at all	4562	88.6
Yes/some ^	586	11.4
**Mothers watching television**		
Not at all	1479	28.7
Yes/some ^	3669	71.3
**Power over household decision making**		
Husband	4480	87.0
Woman alone	667	13.0
**Power over earning**		
Husband	4658	90.5
Woman alone	490	9.5
Birth order		
1st born	4437	86.2
2nd–4th	711	13.8
**Preceding birth interval**		
No previous birth	4471	86.9
Yes	677	13.2
**Sex of baby**		
Male	2621	50.9
Female	2527	49.1
**Age of child (months)**		
0–5	1862	36.2
6–11	1544	30.0
12–17	1088	21.1
18–23	654	12.7
**Low birth weight (<2500 g, *n* = 4464)**		
No	3554	79.6
Yes	910	20.4
**Size of the baby (*n* = 5090)**		
Small	735	14.4
Average	3192	62.7
Large	1164	22.9
**Children who had diarrhea recently**		
No	4452	86.5
Yes	696	13.5
**Children with acute respiratory infection treatment**		
No	4860	94.4
Yes	288	5.6
**Children who had fever in last 2 weeks**		
No	4322	84.0
Yes	826	16.1
***Household-level factors***		
**Wealth Index**		
Poor	2941	57.1
Middle	1236	24.0
Rich	971	18.9
**Number of living children**		
1	4525	87.9
2 to 3	623	12.1
**Source of drinking water**		
Improved	3970	77.1
Unimproved	1178	22.9
**Type of toilet facility**		
Improved	1887	36.7
Unimproved	3261	63.3
***Community-level factors***		
**Residence**		
Urban	995	19.3
Rural	4153	80.7
**Geographical region**		
North	389	7.6
South	910	17.7
East	2066	40.1
West	707	13.7
Central	799	15.5
Northeast	276	5.4
**Type of caste or tribe**		
Schedule caste	1211	23.5
Schedule tribe	699	13.6
Other backward caste	1990	38.7
Other *	1248	24.3

^&^ friend/relative and others; ^#^ divorced/separated/widowed. ^ less than once a week/at least once a week/almost every day; * includes Jews, Parsis/Zoroastrians, those following other religions, and those with no religion. Total count equal to 5148 unless otherwise given in brackets.

**Table 2:** Breastfeeding indicators among children 0–23 months of age, India.

Indicator	Size of Subsample (Weighted)	*n* (Weighted)	Rate (%)	95% CI	
Early initiation of breastfeeding ^a^					
Yes	5148	2256	43.8	41.8	45.9
Ever breastfed rate ^b^					
Yes	5148	5049	98.1	97.5	98.5
Bottle feeding rate ^b^					
Yes	5148	854.1	16.6	15.1	18.3
Exclusive breastfeeding rate ^c^					
Yes	1862	1154	62.0	58.7	65.2
Predominant breastfeeding rate ^c^					
Yes	1862	331.4	17.8	15.5	20.4
Continued breastfeeding rate (1 year) ^d^					
Yes	766.3	676.3	88.3	85.0	90.9
Continued breastfeeding rate (2 years) ^e^					
Yes	408.5	291.4	71.3	62.0	79.1
Early introduction of complementary feeding rate ^f^					
Yes	857	370.9	43.3	38.0	48.7
Minimum dietary diversity ^g^					
Yes	3286	545.6	16.6	14.8	18.7
Minimum meal frequency ^g^					
Yes	3286	898.8	27.4	25.1	29.8
Minimum acceptable diet ^g^					
Yes	3286	222.1	6.8	5.5	8.2
Median duration of any breastfeeding * (months)				4.6	
Median duration of exclusive breastfeeding * (months)				23.0	

^a^ infants under 24 h; ^b^ children under 24 months; ^c^ infants below 6 months; ^d^ children 12–15 months; ^e^ children 20–23 months; ^f^ infants 6–9 months; ^g^ 6–23 months; * children < 36 months, CI-Confidence Interval.

**Table 3:** Factors associated with early initiation of breastfeeding and exclusive breastfeeding in adolescent Indian mothers, 2015–2016 NFHS.

	Early Initiation of Breastfeeding				Exclusive Breastfeeding			
	uOR (95% CI)	*p*	OR (95% CI)	*p*	uOR (95% CI)	*p*	OR (95% CI)	*p*
***Individual-level factors***								
**Mother’s religion**								
Hindu	1.00				1.00			
Muslim	0.97 (0.78, 1.20)	0.766			0.96 (0.68, 1.37)	0.829		
Christian	1.13 (0.73, 1.74)	0.592			1.06 (0.46, 2.45)	0.886		
**Mother’s working status**								
Nonworking	1.00				1.00			
Working (past 12 months)	0.81 (0.41, 1.58)	0.533			6.49 (0.85, 49.81)	0.072		
**Mother’s education**								
No education	1.00				1.00		1.00	
Primary	0.99 (0.74, 1.32)	0.929			0.53 (0.33, 0.86)	0.011	0.55 (0.34, 0.88)	0.013
Secondary and above	1.16 (0.95, 1.43)	0.150			0.65 (0.46, 0.94)	0.022	0.60 (0.41, 0.89)	0.010
**Total Maternal BMI (Kg/m^2^)**								
<18.5	1.00				1.00			
18.5–24.9	1.02 (0.85, 1.21)	0.849			1.27 (0.92, 1.74)	0.144		
25+	0.93 (0.59, 1.45)	0.737			0.84 (0.41, 1.70)	0.620		
**Mother’s age (years)**								
15–17	1.00				1.00			
18	0.77 (0.57, 1.02)	0.070			1.24 (0.81, 1.89)	0.326		
19	0.81 (0.62, 1.06)	0.131			0.86 (0.58, 1.28)	0.459		
**Marital status**								
Currently married	1.00				1.00			
Formerly married ^#^	5.64 (2.69, 11.85)	<0.001			0.27 (0.06, 1.16)	0.079		
**Combined mode and place of delivery**								
Non-caesarean and Home	1.00		1.00		1.00			
Non-caesarean and health facility	1.48 (1.18, 1.86)	0.001	1.36 (1.06, 1.73)	0.013	0.86 (0.60, 1.25)	0.444		
Caesarean and health facility	0.56 (0.41, 0.76)	<0.001	0.43 (0.30, 0.62)	<0.001	0.72 (0.45, 1.18)	0.195		
**Type of delivery assistance**								
Health professional	1.00				1.00			
Traditional birth attendant	0.69 (0.52, 0.92)	0.010			0.90 (0.59, 1.38)	0.641		
Others ^&^	0.68 (0.55, 0.85)	<0.001			0.74 (0.53, 1.05)	0.096		
**Number of antenatal clinic visits**								
8+	1.00		1.00		1.00			
4–7	0.75 (0.56, 1.01)	0.056	0.82 (0.60, 1.11)	0.209	1.33 (0.81, 2.18)	0.265		
1–3	0.64 (0.48, 0.85)	0.002	0.75 (0.55, 1.01)	0.066	0.98 (0.61, 1.58)	0.938		
None	0.52 (0.37, 0.72)	<0.001	0.58 (0.41, 0.83)	0.003	1.06 (0.62, 1.81)	0.836		
**Timely postnatal check**								
0–2 days	1.00				1.00		1.00	
After 2 days	0.64 (0.35, 1.17)	0.147			3.60 (1.25, 10.36)	0.017	3.47 (1.19, 10.08)	0.022
No postnatal check	0.66 (0.42, 1.05)	0.078			2.89 (1.35, 6.18)	0.006	3.06 (1.44, 6.49)	0.003
**Mothers reading newspapers**								
Not at all	1.00				1.00			
Yes/some ^	1.01 (0.84, 1.21)	0.894			0.89 (0.66, 1.18)	0.411		
**Mothers listening to radio**								
Not at all	1.00				1.00			
Yes/some ^	0.96 (0.75, 1.23)	0.746			0.75 (0.51, 1.12)	0.162		
**Mothers watching television**								
Not at all	1.00				1.00			
Yes/some ^	1.17 (0.99, 1.38)	0.068			1.07 (0.81, 1.41)	0.623		
**Power over household decision making**								
Husband	1.00				1.00			
Woman alone	0.98 (0.76, 1.27)	0.896			1.16 (0.73, 1.84)	0.527		
**Power over earning**								
Husband	1.00				1.00			
Woman alone	1.05 (0.78, 1.41)	0.754			1.35 (0.83, 2.18)	0.221		
**Father’s occupation**								
Nonagricultural	1.00				1.00			
Agricultural	1.28 (0.84, 1.96)	0.256			1.19 (0.53, 2.65)	0.677		
Not working	1.19 (0.89, 1.60)	0.238			0.84 (0.48, 1.47)	0.552		
**Birth order**								
1st born	1.00				1.00		1.00	
2nd–4th	1.41 (1.12, 1.77)	0.003			0.49 (0.34, 0.71)	<0.001	0.46 (0.31, 0.66)	<0.001
**Preceding birth interval**								
No previous birth	1.00				1.00			
Yes	1.36 (1.08, 1.71)	0.008			0.5 (0.35, 0.71)	<0.001		
**Sex of baby**								
Male	1.00				1.00			
Female	1.15 (0.97, 1.36)	0.097			0.88 (0.67, 1.16)	0.375		
**Child’s age (in <6 months)**					0.64 (0.59, 0.71)	<0.001		
**Child’s age in category**								
0–5	1.00							
6–11	1.01 (0.82, 1.24)	0.943						
12–17	1.12 (0.90, 1.38)	0.320						
18–23	1.13 (0.85, 1.49)	0.412						
**Low Birth Weight (<2500 g)**								
No	1.00				1.00			
Yes	0.98 (0.79, 1.23)	0.881			1.28 (0.89, 1.82)	0.178		
**Size of the baby**								
Small	1.00				1.00			
Average	0.94 (0.74, 1.19)	0.598			0.92 (0.63, 1.36)	0.688		
Large	0.85 (0.64, 1.13)	0.259			0.84 (0.52, 1.35)	0.469		
**Children who had diarrhea recently**								
No	1.00		1.00		1.00		1.00	
Yes	0.68 (0.54, 0.86)	0.001	0.75 (0.57, 0.97)	0.030	0.58 (0.38, 0.88)	0.012	0.62 (0.40, 0.95)	0.031
**Children with acute respiratory infection treatment**								
No	1.00				1.00			
Yes	0.74 (0.51, 1.06)	0.102			0.42 (0.19, 0.95)	0.037		
**Children who had fever in last 2 weeks**								
No	1.00		1.00		1.00			
Yes	0.72 (0.57, 0.91)	0.006	0.77 (0.61, 0.98)	0.037	0.84 (0.57, 1.24)	0.390		
***Household-level factors***								
**Wealth index**								
Poor	1.00				1.00			
Middle	0.90 (0.74, 1.11)	0.332			0.94 (0.67, 1.32)	0.724		
Rich	0.89 (0.71, 1.12)	0.309			0.86 (0.60, 1.25)	0.435		
**Number of living children**								
1	1.00							
2 to 3	1.51 (1.19, 1.93)	0.001			0.44 (0.30, 0.64)	<0.001		
**Source of drinking water**								
Improved	1.00				1.00			
Unimproved	0.96 (0.79, 1.16)	0.687			1.36 (0.99, 1.86)	0.055		
**Type of toilet facility**								
Improved	1.00				1.00			
Unimproved	1.00 (0.84, 1.20)	0.944			1.3 (0.97, 1.74)	0.085		
***Community-level factors***								
**Residence**								
Urban	1.00				1.00			
Rural	0.87 (0.68, 1.11)	0.264			0.98 (0.67, 1.45)	0.952		
**Geographical region**								
North	1.00		1.00		1.00		1.00	
South	1.84 (1.37, 2.49)	<0.001	2.19 (1.56, 3.06)	<0.001	1.15 (0.70, 1.88)	0.575	1.28 (0.77, 2.11)	0.326
East	1.54 (1.20, 1.99)	0.001	1.69 (1.31, 2.18)	<0.001	0.86 (0.56, 1.31)	0.500	0.82 (0.54, 1.24)	0.360
West	2.55 (1.80, 3.61)	<0.001	2.66 (1.88, 3.75)	<0.001	0.90 (0.50, 1.59)	0.720	0.89 (0.51, 1.57)	0.704
Central	0.90 (0.69, 1.16)	0.430	0.99 (0.76, 1.30)	0.992	0.63 (0.42, 0.96)	0.034	0.63 (0.42, 0.95)	0.031
Northeast	3.92 (2.94, 5.23)	<0.001	4.26 (3.15, 5.75)	<0.001	1.40 (0.85, 2.31)	0.174	1.33 (0.78, 2.25)	0.288
**Type of caste or tribe**								
Schedule caste	1.00				1.00		1.00	
Schedule tribe	0.95 (0.73, 1.23)	0.684			1.98 (1.26, 3.09)	0.003	2.00 (1.27, 3.16)	0.003
Other backward caste	0.87 (0.70, 1.07)	0.183			1.05 (0.73, 1.49)	0.781	1.02 (0.71, 1.45)	0.900
Other *	1.11 (0.85, 1.44)	0.444			1.11 (0.72, 1.70)	0.621	1.08 (0.69, 1.67)	0.727

uOR: unadjusted odds ratio; OR: adjusted odds ratio; CI: confidence interval; *p*: *p*-value; ^&^ friend/relative and others; ^#^ divorced/separated/widowed. ^ less than once a week/at least once a week/almost every day; * includes Jews, Parsis/Zoroastrians, those following other religions, and those with no religion.

**Table 4:** Factors associated with bottle feeding and predominant breastfeeding in adolescent Indian mothers, 2015–2016 NFHS.

	Bottle Feeding				Predominant Breastfeeding			
	uOR (95% CI)	*p*	OR (95% CI)	*p*	uOR (95% CI)	*p*	OR (95% CI)	*p*
***Individual-level factors***								
**Mother’s religion**								
Hindu	1.00				1.00			
Muslim	1.10 (0.82, 1.47)	0.522			0.56 (0.35, 0.90)	0.016		
Christian	0.93 (0.53, 1.63)	0.796			1.25 (0.42, 3.75)	0.685		
**Mother’s working status**								
Nonworking	1.00				1.00			
Working (past 12 months)	0.78 (0.36, 1.69)	0. 583			0.42 (0.05, 3.77)	0.434		
**Mother’s education**								
No education	1.00				1.00		1.00	
Primary	1.40 (0.93, 2.13)	0.110			1.81 (1.06, 3.09)	0.028	1.90 (1.06, 3.39)	0.029
Secondary and above	1.54 (1.15, 2.07)	0.004			1.18 (0.77, 1.80)	0.445	1.41 (0.88, 2.26)	0.142
**Total maternal BMI (Kg/m^2^)**								
<18.5	1.00				1.00			
18.5–24.9	1.04 (0.81, 1.33)	0.748			0.82 (0.57, 1.17)	0.276		
25+	2.14 (1.18, 3.86)	0.012			0.54 (0.23, 1.29)	0.165		
**Mother’s age (years)**								
15–17	1.00				1.00			
18	1.06 (0.69, 1.63)	0.797			1.31 (0.81, 2.14)	0.276		
19	1.24 (0.84, 1.83)	0.286			1.50 (0.94, 2.41)	0.092		
**Marital status**								
Currently married	1.00				1.00			
Formerly married ^#^	0.94 (0.41, 2.19)	0.895			1.91(0.33, 11.10)	0.472		
**Combined mode and place of delivery**								
Non-caesarean and home	1.00		1.00		1.00			
Non-caesarean and health facility	1.72 (1.22, 2.43)	0.002	1.58 (1.10, 2.26)	0.011	1.29 (0.83, 2)	0.259		
Caesarean and health facility	2.98 (1.94, 4.59)	<0.001	2.45 (1.58, 3.81)	<0.001	1.02 (0.56, 1.86)	0.947		
**Type of delivery assistance**								
Health professional	1.00				1.00		1.00	
Traditional birth attendant	0.76 (0.52, 1.1)	0.142			1.46 (0.90, 2.36)	0.122	1.60 (0.94, 2.73)	0.081
Others ^&^	0.84 (0.6, 1.18)	0.321			1.74 (1.13, 2.69)	0.011	1.60 (1.04, 2.47)	0.031
**Number of antenatal clinic visits**								
8+	1.00				1.00			
4–7	0.92 (0.64, 1.33)	0.676			0.73 (0.39, 1.36)	0.318		
1–3	0.92 (0.65, 1.31)	0.655			1.16 (0.64, 2.10)	0.635		
None	0.94 (0.59, 1.51)	0.799			0.96 (0.50, 1.86)	0.907		
**Timely postnatal check**								
0–2 days	1.00				1.00			
After 2 days	0.99 (0.44, 2.20)	0.977			0.79 (0.20, 3.20)	0.742		
No postnatal check	0.84 (0.45, 1.58)	0.596			1.97 (0.64, 6.07)	0.238		
**Mothers reading newspapers**								
Not at all	1.00				1.00			
Yes/some ^	1.03 (0.81, 1.32)	0.809			1.33 (0.93, 1.90)	0.119		
**Mothers listening to radio**								
Not at all	1.00		1.00		1.00			
Yes/some ^	1.49 (1.07, 2.08)	0.018	1.51 (1.07, 2.11)	0.016	0.79 (0.48, 1.30)	0.352		
**Mothers watching television**								
Not at all	1.00				1.00			
Yes/some ^	1.53 (1.20, 1.95)	0.001			0.74 (0.53, 1.03)	0.070		
**Power over household decision making**								
Husband	1.00				1.00			
Woman alone	1.31 (0.89, 1.92)	0.170			1.01 (0.53, 1.91)	0.972		
**Power over earning**								
Husband	1.00				1.00			
Woman alone	1.25 (0.78, 1.99)	0.359			0.99 (0.56, 1.76)	0.980		
**Father’s occupation**								
Nonagricultural	1.00				1.00			
Agricultural	0.53 (0.28, 0.98)	0.043			0.97 (0.33, 2.84)	0.958		
Not working	0.70 (0.45, 1.09)	0.112			1.10 (0.50, 2.44)	0.809		
**Birth order**								
1st born	1.00				1.00			
2nd–4th	0.78 (0.55, 1.10)	0.158			0.98 (0.62, 1.53)	0.912		
**Preceding birth interval**								
No previous birth	1.00				1.00			
Yes	0.66 (0.46, 0.95)	0.025			0.89 (0.56, 1.40)	0.611		
**Sex of baby**								
Male	1.00				1.00			
Female	1.00 (0.80, 1.27)	0.975			1.19 (0.85, 1.66)	0.304		
**Child’s age (in <6 months)**					1.50 (1.34, 1.67)	<0.001	1.51 (1.35, 1.69)	<0.001
**Child’s age in category**								
0–5	1.00		1.00					
6–11	1.65 (1.21, 2.27)	0.002	1.64 (1.20, 2.24)	0.002				
12–17	2.01 (1.48, 2.72)	<0.001	2.07 (1.52, 2.80)	<0.001				
18–23	1.86 (1.28, 2.69)	0.001	1.89 (1.30, 2.75)	0.001				
**Low birth weight (<2500 g)**								
No	1.00				1.00			
Yes	1.16 (0.88, 1.52)	0.286			1.02 (0.67, 1.54)	0.931		
**Size of the baby**								
Small	1.00				1.00			
Average	0.78 (0.58, 1.04)	0.093			1.27 (0.82, 1.98)	0.283		
Large	1.14 (0.79, 1.64)	0.474			0.96 (0.52, 1.76)	0.901		
**Children who had diarrhea recently**								
No	1.00				1.00			
Yes	1.19 (0.89, 1.59)	0.233			1.76 (1.04, 2.96)	0.035		
**Children with acute respiratory infection treatment**								
No	1.00				1.00			
Yes	1.37 (0.89, 2.09)	0.149			1.60 (0.54, 4.81)	0.347		
**Children who had fever in last 2 weeks**								
No	1.00		1.00		1.00			
Yes	1.68 (1.28, 2.20)	<0.001	1.54 (1.17, 2.03)	0.002	1.25 (0.78, 2.01)	0.362		
***Household-level factors***								
**Wealth index**								
Poor	1.00		1.00		1.00			
Middle	1.75 (1.31, 2.33)	<0.001	1.42 (1.08, 1.88)	0.012	1.29 (0.86, 1.94)	0.215		
Rich	1.98 (1.49, 2.64)	<0.001	1.61 (1.20, 2.16)	0.001	1.03 (0.64, 1.65)	0.918		
**Number of living children**								
1	1.00				1.00			
2 to 3	0.76 (0.53, 1.11)	0.160			1.03 (0.65, 1.65)	0.886		
**Source of drinking water**								
Improved	1.00				1.00			
Unimproved	0.85 (0.65, 1.12)	0.245			0.69 (0.47, 1.01)	0.056		
**Type of toilet facility**								
Improved	1.00				1.00			
Unimproved	0.68 (0.53, 0.86)	0.002			0.88 (0.61, 1.28)	0.510		
***Community-level factors***								
**Residence**								
Urban	1.00				1.00			
Rural	0.77 (0.55, 1.07)	0.117			0.99 (0.60, 1.63)	0.977		
**Geographical region**								
North	1.00				1.00		1.00	
South	1.20 (0.82, 1.74)	0.352			0.61 (0.33, 1.15)	0.129	0.63 (0.33, 1.21)	0.168
East	1.06 (0.76, 1.46)	0.747			0.69 (0.42, 1.15)	0.164	0.58 (0.34, 1.00)	0.052
West	0.70 (0.40, 1.23)	0.213			1.37 (0.71, 2.65)	0.345	1.24 (0.65, 2.35)	0.503
Central	1.10 (0.80, 1.52)	0.559			1.51 (0.93, 2.44)	0.088	1.36 (0.82, 2.27)	0.227
Northeast	0.69 (0.48, 1.00)	0.051			0.39 (0.21, 0.75)	0.005	0.31 (0.15, 0.63)	0.001
**Type of caste or tribe**								
Schedule caste	1.00		1.00		1.00			
Schedule tribe	0.52 (0.35, 0.77)	0.001	0.61 (0.40, 0.92)	0.021	0.80 (0.47, 1.37)	0.421		
Other backward caste	1.12 (0.84, 1.51)	0.419	1.05 (0.78, 1.42)	0.727	1.03 (0.66, 1.59)	0.906		
Other *	1.46 (1.02, 2.09)	0.035	1.37 (0.96, 1.96)	0.081	0.61 (0.36, 1.05)	0.074		

uOR: unadjusted odds ratio; OR: adjusted odds ratio; CI: confidence interval; *p*: *p*-value; ^&^ friend/relative and others; ^#^ divorced/separated/widowed. ^ less than once a week/at least once a week/almost every day; * includes Jews, Parsis/Zoroastrians, those following other religions, and those with no religion.

**Table 5:** Factors associated with introduction to solid, semisolid, or soft foods and minimum dietary diversity in adolescent Indian mothers, 2015–2016 NFHS.

	Introduction to Solid, Semisolid or Soft Foods				Minimum Dietary Diversity			
	uOR (95% CI)	*p*	OR (95% CI)	*p*	uOR (95% CI)	*p*	OR (95% CI)	*p*
***Individual-level factors***								
**Mother’s religion**								
Hindu	1.00				1.00			
Muslim	0.81 (0.46, 1.44)	0.477			1.22 (0.86, 1.73)	0.275		
Christian	1.43 (0.56, 3.64)	0.458			1.04 (0.56, 1.93)	0.901		
**Mother’s working status**								
Nonworking	1.00				1.00			
Working (past 12 months)	0.68 (0.15, 3.15)	0.625			0.85 (0.36, 2.05)	0.727		
**Mother’s education**								
No education	1.00				1.00		1.00	
Primary	1.61 (0.79, 3.27)	0.187			1.19 (0.70, 2.01)	0.503	1.20 (0.69, 2.10)	0.504
Secondary and above	1.55 (0.95, 2.52)	0.081			1.86 (1.28, 2.73)	0.001	1.67 (1.10, 2.52)	0.014
**Total maternal BMI (Kg/m^2^)**								
<18.5	1.00				1.00			
18.5–24.9	0.80 (0.52, 1.24)	0.318			0.99 (0.73, 1.34)	0.935		
25+	1.52 (0.46, 5.01)	0.489			1.22 (0.65, 2.30)	0.541		
**Mother’s age (years)**								
15–17	1.00				1.00			
18	0.79 (0.37, 1.70)	0.550			0.97 (0.59, 1.58)	0.897		
19	0.73 (0.37, 1.44)	0.358			1.08 (0.70, 1.67)	0.730		
**Marital status**								
Currently married	1.00				1.00		1.00	
Formerly married ^#^	1.39 (0.17, 1.52)	0.757			0.20 (0.07, 0.56)	0.002	0.19 (0.06, 0.60)	0.005
**Combined mode and place of delivery**								
Non-caesarean and Home	1.00				1.00			
Non-caesarean and Health Facility	0.99 (0.56, 1.73)	0.963			1.34 (0.88, 2.04)	0.171		
Caesarean and Health Facility	1.25 (0.57, 2.74)	0.584			1.95 (1.18, 3.21)	0.009		
**Type of delivery assistance**								
Health professional	1.00				1.00			
Traditional birth attendant	0.87 (0.45, 1.70)	0.682			0.58 (0.36, 0.93)	0.025		
Others ^&^	1.13 (0.62, 2.06)	0.690			1.06 (0.73, 1.56)	0.749		
**Number of antenatal clinic visits**								
8+	1.00		1.00		1.00		1.00	
4–7	0.76 (0.36, 1.60)	0.479	0.88 (0.42, 1.86)	0.752	0.60 (0.38, 0.93)	0.024	0.55 (0.33, 0.90)	0.020
1–3	0.36 (0.17, 0.74)	0.006	0.42 (0.19, 0.91)	0.029	0.47 (0.30, 0.73)	0.001	0.49 (0.29, 0.82)	0.007
None	0.46 (0.17, 1.23)	0.123	0.52 (0.21, 1.30)	0.165	0.41 (0.24, 0.69)	0.001	0.47 (0.26, 0.84)	0.011
**Timely postnatal check**								
0–2 days	1.00				1.00			
After 2 days	0.86 (0.20, 3.77)	0.839			1.09 (0.44, 2.74)	0.847		
No postnatal check	0.51 (0.16, 1.61)	0.251			1.05 (0.52, 2.09)	0.899		
**Mothers reading newspapers**								
Not at all	1.00				1.00			
Yes/some ^	1.30 (0.80, 2.11)	0.296			1.05 (0.78, 1.43)	0.744		
**Mothers listening to radio**								
Not at all	1.00				1.00			
Yes/some ^	1.19 (0.60, 2.38)	0.619			1.27 (0.86, 1.88)	0.230		
**Mothers watching television**								
Not at all	1.00				1.00			
Yes/some ^	1.49 (0.97, 2.27)	0.066			1.67 (1.24, 2.25)	0.001		
**Power over household decision making**								
Husband	1.00							
Woman alone	2.12 (1.02, 4.38)	0.044			1.04 (0.68, 1.57)	0.864		
**Power over earning**								
Husband	1.00				1.00			
Woman alone	1.90 (0.78, 4.63)	0.158			1.12 (0.7, 1.80)	0.628		
**Father’s occupation**								
Nonagricultural	1.00				1.00			
Agricultural	0.61 (0.21, 1.81)	0.370			0.76 (0.39, 1.50)	0.435		
Not working	0.56 (0.24, 1.30)	0.176			1.11 (0.69, 1.78)	0.681		
**Birth order**								
1st born	1.00				1.00			
2nd–4th	1.62 (0.89, 2.95)	0.114			1.08 (0.73, 1.61)	0.701		
**Preceding birth interval**								
No previous birth	1.00				1.00			
Yes	1.57 (0.85, 2.90)	0.149			1.13 (0.76, 1.69)	0.535		
**Sex of baby**								
Male	1.00				1.00			
Female	1.35 (0.88, 2.08)	0.171			1.10 (0.83, 1.46)	0.487		
**Child age in 6–8 months**	1.26 (0.98, 1.62)	0.074						
**Child age in category**					1.00		1.00	
6–11					3.88 (2.59, 5.82)	<0.001	3.95 (2.62, 5.95)	<0.001
12–17					4.64 (3.01, 7.15)	<0.001	4.79 (3.10, 7.42)	<0.001
18–23								
**Low birth weight (<2500 g)**								
No	1.00				1.00			
Yes	0.82 (0.47, 1.44)	0.490			1.29 (0.87, 1.90)	0.209		
**Size of the baby**								
Small	1				1			
Average	0.67 (0.37, 1.23)	0.197			0.75 (0.51, 1.12)	0.156		
Large	0.97 (0.46, 2.02)	0.929			1.05 (0.67, 1.65)	0.841		
**Children who had diarrhea recently**								
No	1.00				1.00			
Yes	1.62 (0.96, 2.74)	0.070			1.03 (0.71, 1.49)	0.879		
**Children with acute respiratory infection treatment**								
No	1.00				1.00			
Yes	1.62 (0.73, 3.61)	0.232			1.06 (0.62, 1.82)	0.836		
**Children who had fever in last 2 weeks**								
No	1.00				1.00		1.00	
Yes	1.31 (0.78, 2.18)	0.306			1.50 (1.05, 2.15)	0.025	1.65 (1.09, 2.48)	0.016
***Household-level factors***								
**Wealth index**								
Poor	1.00		1.00		1.00			
Middle	2.68 (1.59, 4.50)	<0.001	2.57 (1.50, 4.40)	0.001	1.11 (0.80, 1.56)	0.525		
Rich	1.10 (0.62, 1.94)	0.735	1.02 (0.54, 1.90)	0.941	1.23 (0.83, 1.82)	0.297		
**Number of living children**								
1	1.00				1.00			
2 to 3	1.78 (0.94, 3.40)	0.079			1.15 (0.75, 1.77)	0.523		
**Source of drinking water**								
Improved	1.00				1.00			
Unimproved	0.91 (0.56, 1.49)	0.710			0.86 (0.61, 1.23)	0.414		
**Type of toilet facility**								
Improved	1.00				1.00			
Unimproved	0.79 (0.50, 1.26)	0.325			0.89 (0.67, 1.20)	0.453		
***Community-level factors***								
**Residence**								
Urban	1				1			
Rural	0.82 (0.42, 1.60)	0.555			0.87 (0.58, 1.29)	0.476		
**Geographical region**								
North	1.00		1.00		1.00		1.00	
South	2.79 (1.25, 6.22)	0.012	2.15 (0.89, 5.18)	0.085	4.41 (2.62, 7.43)	<0.001	3.59 (2.02, 6.40)	<0.001
East	1.58 (0.83, 3.00)	0.160	1.69 (0.85, 3.36)	0.133	2.73 (1.67, 4.44)	<0.001	2.77 (1.68, 4.56)	<0.001
West	3.37 (1.36, 8.38)	0.009	2.66 (1.04, 6.76)	0.040	1.47 (0.70, 3.07)	0.299	1.22 (0.55, 2.71)	0.622
Central	2.22 (1.18, 4.17)	0.012	2.71 (1.37, 5.35)	0.004	1.14 (0.67, 1.93)	0.610	1.29 (0.75, 2.24)	0.346
Northeast	2.27 (1.07, 4.78)	0.031	2.63 (1.18, 5.87)	0.018	3.13 (1.88, 5.22)	<0.001	3.38 (1.91, 5.98)	<0.001
**Type of caste or tribe**								
Schedule caste	1.00				1.00		1.00	
Schedule tribe	1.03 (0.57, 1.89)	0.916			0.57 (0.37, 0.88)	0.012	0.85 (0.52, 1.39)	0.534
Other backward caste	0.84 (0.50, 1.43)	0.531			0.67 (0.46, 0.96)	0.031	0.64 (0.42, 0.98)	0.040
Other *	1.22 (0.62, 2.39)	0.571			0.67 (0.43, 1.03)	0.070	0.59 (0.34, 1.02)	0.060

uOR: unadjusted odds ratio; OR: adjusted odds ratio; CI: confidence interval; *p*: *p*-value; ^&^ friend/relative and others; ^#^ divorced/separated/widowed. ^ less than once a week/at least once a week/almost every day; * includes Jews, Parsis/Zoroastrians, those following other religions, and those with no religion.

**Table 6:** Factors associated with minimum meal frequency and minimum acceptable diet in adolescent Indian mothers, 2015–2016 NFHS.

	Minimum Meal Frequency				Minimum Acceptable Diet			
	uOR (95% CI)	*p*	OR (95% CI)	*p*	uOR (95% CI)	*p*	OR (95% CI)	*p*
***Individual-level factors***								
**Mother’s religion**								
Hindu	1.00				1.00			
Muslim	0.91 (0.67, 1.25)	0.560			0.8 (0.42, 1.52)	0.491		
Christian	1.37 (0.79, 2.40)	0.266			1.11 (0.42, 2.96)	0.837		
**Mother’s working status**								
Nonworking	1.00				1.00			
Working (past 12 months)	1.08 (0.54, 2.16)	0.825			0.45 (0.14, 1.41)	0.173		
**Mother’s education**								
No education	1.00		1.00		1.00		1.00	
Primary	1.51 (1.00, 2.28)	0.046	1.59 (1.06, 2.41)	0.025	1.45 (0.63, 3.30)	0.374	1.52 (0.66, 3.49)	0.318
Secondary and above	1.25 (0.95, 1.65)	0.105	1.41 (1.06, 1.87)	0.016	1.83 (1.01, 3.31)	0.045	1.94 (1.06, 3.53)	0.030
**Total maternal BMI (Kg/m^2^)**								
<18.5	1.00				1.00			
18.5–24.9	1.06 (0.84, 1.35)	0.613			0.81 (0.52, 1.29)	0.377		
25+	1.38 (0.67, 2.85)	0.382			0.61 (0.22, 1.74)	0.355		
**Mother’s age (years)**								
15–17	1.00		1.00		1.00			
18	1.54 (0.99, 2.41)	0.054	1.60 (1.02, 2.51)	0.039	1.24 (0.59, 2.61)	0.573		
19	1.51 (1.00, 2.28)	0.047	1.57 (1.04, 2.37)	0.030	1.18 (0.59, 2.33)	0.642		
**Marital status**								
Currently married	1.00				1.00		1.00	
Formerly married ^#^	1.98 (0.72, 5.48)	0.187			0.11 (0.03, 0.38)	0.001	0.12 (0.03, 0.44)	0.001
**Combined mode and place of delivery**								
Non-caesarean and home	1.00				1.00			
Non-caesarean and health facility	1.21 (0.88, 1.66)	0.241			1.37 (0.66, 2.88)	0.401		
Caesarean and health facility	1.39 (0.89, 2.18)	0.152			1.91 (0.82, 4.47)	0.134		
**Type of delivery assistance**								
Health professional	1.00				1.00			
Traditional birth attendant	0.82 (0.57, 1.2)	0.318			0.61 (0.33, 1.15)	0.128		
Others ^&^	1.30 (0.96, 1.76)	0.092			1.19 (0.71, 2.01)	0.510		
**Number of antenatal clinic visits**								
8+	1.00				1.00			
4–7	1.42 (0.96, 2.1)	0.075			1.11 (0.58, 2.13)	0.750		
1–3	0.80 (0.55, 1.18)	0.263			0.55 (0.28, 1.07)	0.078		
None	1.06 (0.66, 1.69)	0.822			0.66 (0.3, 1.46)	0.306		
**Timely postnatal check**								
0–2 days	1.00				1.00			
After 2 days	1.87 (0.72, 4.85)	0.195			2.97 (0.6, 14.75)	0.183		
No postnatal check	1.72 (0.78, 3.83)	0.177			3.10 (1.02, 9.45)	0.047		
**Mothers reading newspapers**								
Not at all	1.00				1.00			
Yes/some ^	1.17 (0.91, 1.52)	0.220			0.87 (0.53, 1.43)	0.585		
**Mothers listening to radio**								
Not at all	1.00				1.00			
Yes/some ^	1.10 (0.79, 1.54)	0.571			1.13 (0.60, 2.12)	0.698		
**Mothers watching television**								
Not at all	1.00				1.00			
Yes/some ^	1.49 (1.18, 1.88)	<0.001			1.47 (0.93, 2.31)	0.099		
**Power over household decision making**								
Husband	1.00				1.00			
Woman alone	1.21 (0.82, 1.78)	0.343			1.42 (0.79, 2.56)	0.247		
**Power over earning**								
Husband	1.00				1.00			
Woman alone	1.18 (0.74, 1.87)	0.485			0.91 (0.49, 1.69)	0.763		
**Father’s occupation**								
Nonagricultural	1.00				1.00			
Agricultural	0.57 (0.32, 1.03)	0.061			0.48 (0.18, 1.24)	0.128		
Not working	0.75 (0.49, 1.16)	0.198			0.78 (0.40, 1.50)	0.449		
**Birth order**								
1st born	1.00				1.00			
2nd–4th	0.83 (0.60, 1.14)	0.247			0.64 (0.31, 1.35)	0.243		
**Preceding birth interval**								
No previous birth	1.00				1.00			
Yes	0.80 (0.58, 1.12)	0.194			0.67 (0.32, 1.41)	0.292		
**Sex of baby**								
Male	1.00				1.00			
Female	0.97 (0.76, 1.22)	0.770			1.11 (0.73, 1.70)	0.597		
**Age of child (months)**								
6–11	1.00				1.00			
12–17	0.86 (0.63, 1.18)	0.350			0.29 (0.16, 0.51)	<0.001		
18–23	0.94 (0.69, 1.28)	0.696			0.69 (0.43, 1.12)	0.134		
**Low Birth Weight (<2500 g)**								
No	1.00				1.00			
Yes	0.62 (0.45, 0.86)	<0.001			1.06 (0.58, 1.93)	0.854		
**Size of the baby**								
Small	1.00				1.00			
Average	0.86 (0.63, 1.18)	0.342			0.51 (0.29, 0.91)	0.022		
Large	0.95 (0.64, 1.42)	0.818			0.66 (0.33, 1.31)	0.234		
**Children who had diarrhea recently**								
No	1.00				1.00			
Yes	1.15 (0.86, 1.54)	0.362			1.06 (0.58, 1.93)	0.854		
**Children with acute respiratory infection treatment**								
No	1.00				1.00			
Yes	0.78 (0.50, 1.23)	0.289			0.70 (0.26, 1.91)	0.489		
**Children who had fever in last 2 weeks**								
No	1.00				1.00			
Yes	0.96 (0.72, 1.28)	0.782			1.21 (0.72, 2.02)	0.471		
***Household-level factors***								
**Wealth index**								
Poor	1.00				1.00			
Middle	1.19 (0.88, 1.61)	0.254			0.87 (0.52, 1.47)	0.609		
Rich	0.92 (0.67, 1.27)	0.610			0.84 (0.47, 1.52)	0.570		
**Number of living children**								
1	1.00				1.00			
2 to 3	0.80 (0.57, 1.14)	0.220			0.70 (0.32, 1.55)	0.381		
**Source of drinking water**								
Improved	1.00				1.00			
Unimproved	1.20 (0.91, 1.58)	0.195			0.84 (0.47, 1.52)	0.567		
**Type of toilet facility**								
Improved	1.00				1.00			
Unimproved	0.99 (0.77, 1.28)	0.962			0.92 (0.60, 1.42)	0.712		
***Community-level factors***								
**Residence**								
Urban	1.00				1.00			
Rural	0.98 (0.68, 1.42)	0.922			1.63 (0.89, 2.98)	0.112		
**Geographical Region**								
North	1.00		1.00		1.00		1.00	
South	1.48 (0.93, 2.34)	0.090	1.45 (0.91, 2.32)	0.111	3.53 (1.46, 8.56)	0.005	3.14 (1.28, 7.68)	0.012
East	1.64 (1.12, 2.40)	0.010	1.69 (1.15, 2.48)	0.007	3.70 (1.63, 8.37)	0.002	3.56 (1.57, 8.07)	0.002
West	1.43 (0.83, 2.46)	0.196	1.39 (0.81, 2.38)	0.223	1.10 (0.38, 3.23)	0.850	1.02 (0.34, 3.09)	0.962
Central	2.02 (1.38, 2.96)	<0.001	2.00 (1.36, 2.94)	<0.001	1.89 (0.80, 4.46)	0.141	1.86 (0.78, 4.39)	0.156
Northeast	1.37 (0.90, 2.10)	0.140	1.39 (0.89, 2.18)	0.145	3.16 (1.33, 7.52)	0.009	3.18 (1.30, 7.76)	0.011
**Type of caste or tribe**								
Schedule caste	1.00		1.00		1.00		1.00	
Schedule tribe	1.08 (0.75, 1.54)	0.673	1.14 (0.79, 1.64)	0.471	0.58 (0.31, 1.06)	0.078	0.74 (0.40, 1.37)	0.344
Other backward caste	0.73 (0.54, 0.98)	0.036	0.73 (0.54, 0.98)	0.037	0.52 (0.31, 0.88)	0.015	0.53 (0.31, 0.89)	0.017
Other *	0.73 (0.50, 1.08)	0.118	0.75 (0.51, 1.10)	0.148	0.54 (0.28, 1.03)	0.062	0.54 (0.27, 1.07)	0.082

uOR: unadjusted odds ratio; OR: adjusted odds ratio; CI: confidence interval; *p*: *p*-value; ^&^ friend/relative and others; ^#^ divorced/separated/widowed. ^ less than once a week/at least once a week/almost every day; * includes Jews, Parsis/Zoroastrians, those following other religions, and those with no religion.

## Supplementary Materials

The following are available online at https://www.mdpi.com/article/10.3390/nu13072376/s1, Table S1: Definition and categorization of potential variables used in the study.

## Abbreviations

IYCF—infant and young child feeding; ANC—antenatal care; SDGs—Sustainable Development Goals; WHO—World Health Organization; BF—breastfeeding; CF—complementary feeding, NFHS—National Family Health Survey; DHS—Demographic and Health Survey LMIC—lower and middle income countries; BMI—Body Mass Index; OR—Adjusted odds ratios; uOR—Unadjusted odds ratio; CI—confidence intervals.
