# Supplementary material for: Infant and Young Child Feeding Practices among Adolescent Mothers and Associated Factors in India
Source: Nutrients. 2021 Jul 12;13(7):2376. doi: 10.3390/nu13072376 (PMC8308797; doi:10.3390/nu13072376)
Supplement: Supplementary file 1 [file nutrients-13-02376-s001.zip › nutrients-1264612-supplementary.pdf]

Supplementary Table S1. Definition and categorisation of potential variables used in the study

Independent variables	Categorisation
Individual-level factors	
Maternal religion	In the 3 following categories: (1=Hindu; 2=Muslim; 3=Christian)
Maternal working status	In the 2 following categories: (1=not working; 2=working (for the past 12 months))
Maternal education	In the 3 following categories: (1=No education; 2=Primary; 3=Secondary or higher)
Literary	In the 2 following categories: (1= No Literacy; 2= Literacy)
Maternal BMI	In the 3 following categories: (1= <18.5kg/m^2; 2= 18.5-24.9 kg/m^2; 3= 25+ kg/m^2)
Adolescent mother's age	In the 3 following categories: (1= 15-17 years; 2= 18 years; 3= 19 years)
Marital status	In the 2 following categories: (1= Currently married; 2=divorced/separated/widow)
Combined mode and place of delivery	In the 3 following categories: (1= Non-Caesarean & Home; 2= Non-Caesarean & Health Facility; 3= Caesarean & Health Facility)
Type of delivery assistance	In the 3 following categories: (1=Health professional[&]; 2= Traditional birth attendant; 3= Others)
Antenatal Clinic visits	In the 4 following categories: (1) ≥8 antenatal care visits, (2) 4-7 antenatal care visits, and (3) 1-3 antenatal care visits (4) no antenatal care visits from a skilled provider for the most recent birth
Postnatal check-up	In the 3 following categories: (1=0-2 days; 2=After 2 days; 3=No postnatal check-up)
Adolescent mother reading newspapers	In the 2 following categories: (1= No; 2=Yes/some)
Adolescent mother listening to radio	In the 2 following categories: (1= No; 2=Yes/some)
Adolescent mother watching television	In the 2 following categories: (1= No; 2=Yes/some)
Decision making	In the 2 following categories:(1= mother involved; 2= mother not involved)
Power over household decision making	In the 2 following categories:(1=Husband; 2= woman alone)
Power over earning	In the 2 following categories:(1=Husband; 2= woman alone)
Father's occupation	In the 3 following categories: (1=Non-agricultural; 2=Agricultural; 3=Not working)
Birth order	In the 2 following categories: (1= First-born; 2=2nd-4th born)
Preceding birth interval	In the 2 following categories: (1= No previous birth; 2=Yes)

Sex of baby	In the 2 following categories: (1=Male; 2=Female)
Age of child (months)	In the 4 following categories: (1=0-5 months; 2=6-11 months; 3=12-17 months; 4=18-23 months)
Low birth weight (<2500gms)	In the 2 following categories: (1= Not at all; 2=Yes)
Size of baby	In the 3 following categories: (1= Small; 2=Average; 3= Large)
Had diarrhoea recently	In the 2 following categories: (1= No; 2=Yes)
Acute respiratory infection treatment	In the 2 following categories: (1= No; 2=Yes)
Had fever in last 2 weeks	In the 2 following categories: (1= No; 2=Yes)
Household-level factors	
Household wealth Index	hv217 (the household wealth index factor score) constructed by DHS based on a selected set of household assets. In quintiles, 1= poor; 2= middle; 3= rich)
Number of living children	In the 2 following categories: (1= 1 child; 2=2-3 children)
Source of drinking water	In the 2 following categories: (1= improved; 2=Unimproved)
Type of toilet facility	In the 2 following categories: (1= improved; 2=Unimproved)
Community-level factors	
Residence	In the 2 following categories:(1=urban; 2=rural)
Geographical Regions	In the 6 following categories: (1=North; 2=South; 3= East; 4= West; 5= Central; 6= Northeast)
Type of caste or tribe	In the 4 following categories (1= Scheduled caste; 2= Schedule tribe; 3= Other backward caste; 4= Others)

&= doctor; nurse/midwife/paramedics, family welfare visitor medical assistant/community medical officer/ health assistant
